# Turkish adaptation of a self-report measure for current achievement: the Current Motivation Questionnaire

**DOI:** 10.3389/fpsyg.2025.1647805

**Published:** 2026-01-08

**Authors:** Sena Seçil Akpınarlı, Pınar Köseoğlu

**Affiliations:** Department of Biology Education, Faculty of Education, Hacettepe University, Ankara, Türkiye

**Keywords:** Current Achievement Motivation, Current Motivation Questionnaire, instrument adaptation, reliability, validity, pre-service teacher

## Abstract

The absence of a valid and reliable instrument to measure situational motivation in the Turkish context highlights the need for adapting the Current Motivation Questionnaire (QCM). This study aims to adapt the short form of the QCM into Turkish and examine its psychometric properties. The study was conducted with 302 pre-service teachers during the 2024–2025 academic year in Ankara, Turkiye. Linguistic validity was examined through translation–back translation and correlation analysis. Construct validity was tested using Confirmatory Factor Analysis (CFA), and reliability was examined using Cronbach’s alpha coefficients, item–total correlations, and top–bottom 27% group comparisons.CFA supported the original four-factor structure (Interest, Anxiety, Probability of Success, Challenge) of the QCM with acceptable model fit indices (χ^2^/df = 2.79, RMSEA = 0.094, CFI = 0.940, TLI = 0.914, GFI = 0.905). Internal consistency was high for the overall questionnaire (α = 0.893), and item discrimination analyses indicated that all items were statistically significant (*p* < 0.001). The findings demonstrate that the Turkish version of the QCM is a valid and reliable instrument for assessing pre-service teachers’ situational motivation. The scale can be effectively used in task-based and mobile-assisted outdoor learning contexts within Turkish educational settings.

## 1 Introduction

“Motivation” is regarded as the most significant factor influencing human behavior and performance in a given situation (Turan, 2015). The word originates from the Latin term “movere,” meaning “to move” or “to set in motion” (Kreitner and Kinichi, 2009). Gottfried (1990) described motivation as a complex construct comprising beliefs, perceptions, values, interests, and actions, all of which are closely interconnected. Being motivated means taking the necessary action to accomplish something (Deci and Ryan, 2000). Various studies emphasize that motivation in the field of education significantly affects a student’s success in a particular subject [Williams and Williams, 2011; Brooks and Goldstein, 2008; Dweck, 2006 as cited in Bayrakçeken et al. (2021)].

**Figure 1:**
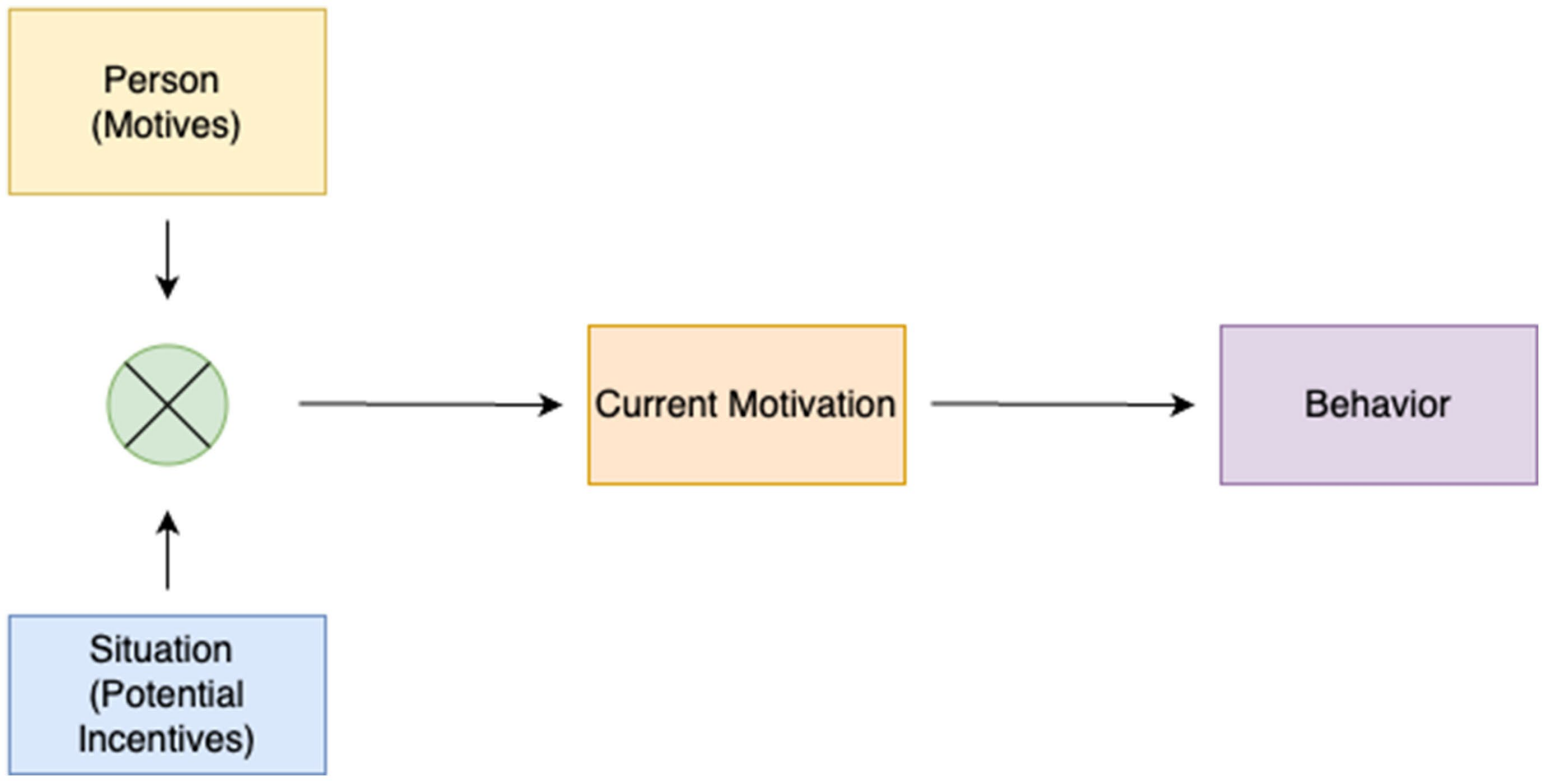
Basic model of classical motivation psychology [adapted from Rheinberg (2000, p. 70)].

In general motivation theories (McClelland et al., 1953; Murray, 1943; Atkinson, 1974), achievement motivation has been addressed and noted to be associated with individuals’ overall performance in achieving success. Achievement motivation is a multidimensional concept encompassing cognitive, emotional, and behavioral dimensions, as well as patterns of individual and social behavior. However, high achievement motivation does not always guarantee the expected behavior will be exhibited. The characteristics and instructions of the task in the process play a highly critical role (Rheinberg et al., 2001). Classical motivation psychology argues that behaviors are shaped by the interaction of individual (motives) and environmental (situational incentives) factors (Lewin, 1946; Atkinson, 1957; Rheinberg et al., 2001). Motives are enduring characteristics that reflect an individual’s interest in specific types of incentives. Situational incentives, on the other hand, provide opportunities that align with an individual’s motives. This alignment triggers the individual’s current motivation, energizing and directing their behavior (Figure 1).

Several self-report instruments have been developed to assess achievement motivation [e.g., Achievement Motivation Profile-AMP, Mandel and Marcus, 1988; Current Motivation Questionnaire-QCM, Rheinberg et al., 2001; Freund et al., 2011; Achievement Motivation Inventory-AMI, Smith, 1972; Smith et al., 2022, as cited in Karaman et al. (2023)]. Among these, the QCM (Rheinberg et al., 2001; Freund et al., 2011) is a measurement tool designed to assess situational achievement motivation factors. According to Rheinberg et al. (2001), situational achievement motivation consists of four factors: interest, anxiety, probability of success, and challenge. The short form of the QCM (Freund et al., 2011) also comprises four factors representing these factors.

Various studies have been conducted using the QCM to measure motivation in learning processes. For example, Bedek et al. (2015) utilized behavioral indicators in digital educational games to predict motivational states with the short form of the QCM and emphasized its validity for online learning applications. Killingsworth and Clark (2013) examined students’ learning processes regarding Newton’s Laws of Motion and the effectiveness of attention networks using various assessment tools. Notably, the short form of the QCM was used to measure motivation and engagement levels. The QCM assessed motivational factors such as anxiety, challenge, interest, and probability of success experienced by students during gameplay. The study demonstrated, in detail, the motivational effects of digital games and their contributions to learning using the adapted version of the QCM.

Elford (2022) investigated the relationship between cognitive load and achievement motivation in augmented reality-supported educational processes using the QCM. Freund et al. (2017), in their study, examined task-specific achievement motivation (Current Achievement Motivation—CAM) across four dimensions (interest, probability of success, perceived difficulty, and fear of failure). They concluded that some aspects of motivation are task-dependent, while others are more stable. Interest and probability of success were found to be influenced by task characteristics, whereas perceived difficulty and fear of failure were relatively stable. The findings of Freund and colleagues indicate that motivational differences provide significant insights into explaining variations in individuals’ task performance.

Alpaslan (2023) examined the impact of storytelling, one of the gamification elements, on motivation and learning outcomes in online Inquiry-Based Learning (IBL) environments. Using the QCM, the study was conducted with higher education students, comparing motivation levels between groups as well as pre- and post-intervention levels of participants. Meyerhöffer and Dreesmann (2021), in their study, developed and tested a content-based video exchange model aimed at introducing English learners at the lower secondary level to English as a scientific language in a motivating way. After field trips to forest and desert ecosystems, students presented and compared biotic and abiotic data in videos. The content knowledge and motivation of German students were assessed using a pre-test/post-test design.

In their study, Kissi and Dreesmann (2022) examined a teaching approach developed to address plant blindness, a common issue in the context of plant education. The “Educational Seed Mix,” consisting of eight perennial species from six different plant families, enabled students aged 10–15 to experience plant diversity, connect with their plants, observe the growth of a plant from seed to flower, and gain knowledge about floral biology in an authentic learning environment. In an evaluation study conducted with secondary school students in Germany, findings obtained through the QCM revealed that students showed high interest prior to the project and experienced a sense of achievement and enthusiasm regarding plant cultivation afterward. Similarly, in another study by Kissi and Dreesmann (2018), an activity was designed by combining mobile learning and plant education to combat plant blindness. To encourage students’ direct interaction with plants, an interactive mobile treasure hunt was developed in the context of a botanical garden. This activity, called the “Flower Hunt,” focused on topics such as flower diversity, morphology, ecology, and systematics. The activity increased students’ motivation and engagement, and although it did not completely eliminate plant blindness, it strengthened their connection to nature and plants. In this context, the use of the QCM in various learning environments facilitates the effective assessment of motivation.

According to Rheinberg et al. (2001), while the long-term effects of individuals’ motivation can be identified in daily life, it is challenging to predict the impact of motivational factors in the short term and in specific situations. Therefore, directly measuring the activated motivation in a particular situation is considered an accurate approach for determining its influence on behavior. In this context, examining individuals’ situational motivation becomes significant. The aim of this study is to adapt the short form of the QCM into Turkish culture to measure the situational motivation of pre-service teachers during task-based outdoor activities involving plants using mobile devices.

## 2 Method

In this study, based on the responses of pre-service teachers to the Current Motivation Questionnaire (QCM), linguistic equivalence was first ensured, followed by analyses to determine the reliability and validity of the scale (Büyüköztürk et al., 2020).

### 2.1 Participants

This study included 70 pre-service teachers who participated in the pilot study and 206 pre-service teachers who participated in the main study, all of whom were enrolled in two different departments of the faculty of education at a university in Ankara, Turkey, and had taken the “general biology” course. However, after identifying the responses of four pre-service teachers as outliers, their data were excluded from the study. Therefore, the main application data were analyzed based on the responses of 202 participants. Additionally, 30 pre-service teachers who were proficient in both English and Turkish participated in the initial linguistic validity study. In total, 302 pre-service teachers participated in all processes of the study. A common characteristic of these participants was that they had taken biology courses during their undergraduate education. The Turkish version of the questionnaire was administered to pre-service teachers from these two departments to measure their situational motivation following instructions on conducting tasks related to plant species outdoors using mobile devices within the framework of task-based learning. This sampling choice aligns with the contextual demands of the QCM, which is designed to measure situational motivation in outdoor, task-based activities relevant to pre-service teacher training curricula. In questionnaire development and adaptation studies, it is suggested that the sample size should be at least five times (Bryman and Cramer, 2002), ten times (Nunnally, 1978), or fifteen times (Gorusch, 1983) the number of items (Delice and Ergene, 2015). Accordingly, the maximum number of pre-service teachers from these two departments was reached to carry out the study based on the specified task instructions. Table 1 presents the descriptive statistics for the participants.

**Table 1:** Descriptive statistics for the participants.

Variable	Category	*f*	%
Gender	Female	146	72.2
	Male	56	27.7
Department	Biology teaching	73	36.1
	Science teaching	129	63.8
Grade level	1st grade	43	21.3
	2nd grade	47	23.2
	3rd grade	50	24.7
	4th grade	62	30.7
Total		202	100

According to Table 1, 72.2% (*N* = 146) of the participants are female, and 27.7% (*N* = 56) are male. Additionally, 36.1% (*N* = 73) are enrolled in biology teaching programs, while 63.8% (*N* = 129) are studying in science teaching programs. Of the participants, 21.3% (*N* = 43) are first-year students, 23.2% (*N* = 47) are second-year students, 24.7% (*N* = 50) are third-year students, and 30.7% (*N* = 62) are fourth-year students.

### 2.2 Current Motivation Questionnaire short form (QCM)

QCM is a questionnaire designed to assess individuals’ situational achievement motivation. The short form of the questionnaire consists of four factors—“Interest,” “Anxiety,” “Challenge,” and “Probability of Success”—and includes a total of 12 items. The items in the questionnaire are rated using a 7-point Likert scale (1 = Strongly Disagree, 7 = Strongly Agree). The short form of QCM was developed through two separate studies by Rheinberg et al. (2001). In the first study, the measurement model of the questionnaire was tested, and a 12-item short form was derived from the original 18-item long form. Data from 350 students who participated in the Latin Square Task were used during this process. In the second study, the measurement properties of the short form were validated using a larger sample of 509 students. During the development of the short form, six items were eliminated, and the results demonstrated that the short form provided psychometrically acceptable fit. Moreover, the item-total correlations and internal consistency values of the short form were found to be higher compared to the original form, highlighting the superior psychometric properties of the questionnaire. The short form, noted for being an economical and practical measurement tool, was repeated in Study 2, further confirming its high quality.

### 2.3 Translation

Permissions were obtained via email from Freund et al. (2011), the authors of the short form of the questionnaire, and Rheinberg et al. (2001), the authors of the original questionnaire, to adapt the questionnaire into Turkish and use it in this study. Once the permission process was completed, the adaptation process began. The first step in the adaptation process was to ensure linguistic validity. To achieve linguistic validity, the questionnaire items were independently translated into Turkish by a specialist in English Language and Literature and a faculty member from the English Language Teaching department. Subsequently, the translations were compared by the researcher and another English Language Teaching faculty member, and the first Turkish version of the questionnaire was finalized. This initial version of the questionnaire was then evaluated by two faculty members from the Psychological Counseling and Guidance department in terms of the meaning and coherence of the translated items. After incorporating the suggested revisions, the Turkish version of the questionnaire was sent to a Turkish language specialist and a faculty member from the Turkish Language Teaching department for evaluation of grammar and comprehensibility. Based on the feedback provided, some synonymous words and grammatical rules in the items were revised. Following these revisions, the questionnaire was back-translated into its original language by two different language specialists. The original version of the questionnaire and the back-translated version were compared by another specialist in English Language Teaching, and a high level of similarity was found. After completing these procedures, the linguistic adaptation process was finalized, and correlation analysis was conducted to examine linguistic validity.

### 2.4 Data analysis

For data analysis, SPSS 26 software was used, while AMOS 23 software was employed for Confirmatory Factor Analysis (CFA). To examine the construct validity of the questionnaire, first-order fit indices such as *X*^2^/df, RMSEA, CFI, TLI, GFI, and AGFI were utilized. For reliability analysis, Cronbach’s alpha internal consistency coefficient was calculated. Seçer (2015) emphasized the necessity of conducting Confirmatory Factor Analysis (CFA) directly instead of Exploratory Factor Analysis (EFA) during the questionnaire adaptation process. Therefore, a researcher aiming to adapt a measurement tool developed abroad into Turkish culture should investigate the compatibility of the existing latent structure with Turkish culture rather than redefining the structure already tested and validated (Seçer, 2015). Accordingly, Confirmatory Factor Analysis (CFA) was conducted to confirm the factor structure of the translated questionnaire with predefined factors (Keith and Reynolds, 2018; Tabachnick and Fidell, 2013). To examine item discrimination, the mean scores of the top and bottom 27% groups were compared. In terms of reliability, Cronbach’s alpha reliability coefficient was calculated for the dimensions and the entire questionnaire.

## 3 Findings

This section presents the findings obtained based on the results of Confirmatory Factor Analyses in the context of validity and reliability analyses, along with linguistic validity.

### 3.1 Testing linguistic validity through correlation analysis

After completing the translation process of the original form into Turkish, statistical methods were employed to examine linguistic validity. To assess the linguistic validity of the questionnaire, it must be administered to a sample group proficient in both Turkish and the original language of the scale (Seçer, 2015). Since the target group of the questionnaire comprised individuals proficient in both languages, an analysis method was utilized instead of revisiting expert opinions. Researchers administered the Turkish version of the questionnaire and the original form published in its original language to a group of 30 participants, with a one-week interval between the two administrations. After the applications, correlation values between the questionnaire forms of the same participants were calculated. In this context, the results of the Pearson Product–Moment Correlation analysis to examine the relationship between the two forms are presented in Table 2.

**Table 2:** Correlations between forms in the original language and forms translated into Turkish.

		Original form	Turkish form
Original form	Pearson correlation	1	0.931^a^
	Sig. (2-tailed)		0.000
	*N*	30	30
Turkish form	Pearson correlation	0.931^a^	1
	Sig. (2-tailed)	0.000	
	N	30	30

a Correlation is significant at the 0.01 level (2-tailed).

According to Table 2, the correlation results between the two forms indicate a strong relationship (*r* = 0.931, *p* < 0.001) and suggest that the two forms measure the same construct. As the correlation value approaches 1.00, the degree of the relationship between the two variables increases, whereas as it approaches 0.00, the degree of the relationship decreases. A high correlation value demonstrates that the two forms are linguistically equivalent and measure the same construct (Seçer, 2015). Based on the findings in Table 2, the high correlation coefficient (0.931) and the significant *p*-value support the consistency of the Turkish version of the questionnaire with the original version in terms of linguistic validity and its effectiveness in measuring the same construct. This result reinforces the accuracy and reliability of the translation, showing that both forms consistently evaluate the same concept or behavior despite linguistic differences.

### 3.2 Pilot study

After ensuring the linguistic validity of the QCM through translation processes, a pilot study was conducted to examine the internal consistency and the compatibility of item-total correlations. The pilot study was carried out with a sample group of 70 participants, focusing on whether the internal consistency value of the QCM exceeded 0.70 and whether the item-total correlation values were below 0.30. Once it was determined that the internal consistency value of the QCM was above 0.70 and the item-total correlation values were above 0.30, validity and reliability analyses of the QCM were conducted.

### 3.3 Descriptive statistics

To assess sample adequacy, the Kaiser-Meyer-Olkin (KMO) coefficient and Bartlett’s Sphericity test results were examined. According to Table 3, the KMO value was found to be 0.877, and the result of Bartlett’s Sphericity test was *p* < 0.001. According to Field (2009), a KMO coefficient greater than 0.50 and a statistically significant Bartlett’s Sphericity test indicate that the data is suitable for factor analysis.

**Table 3:** The KMO and Bartlett’s sphericity test results for QCM adapted to Turkish.

KMO measure of sampling adequacy	Approx. *X*^2^	df	Sig.
0.877	1409.564	66	0.000

### 3.4 Construct validity

Confirmatory Factor Analysis (CFA) is a factor analysis approach frequently used to examine the model fit of the latent structure obtained through Exploratory Factor Analysis during the adaptation process of a measurement tool developed abroad into Turkish or during the development of an original measurement tool (Seçer, 2015). Meydan and Şeşen (2011) define CFA as a method that allows researchers to test whether the data they have fits a previously discovered original structure. CFA was conducted using the SPSS AMOS 23 software to support the four-factor structure of the questionnaire.

To test the four-dimensional structure of the QCM, Confirmatory Factor Analysis (CFA) and the Maximum Likelihood estimation method were used. After selecting the estimation method, the fit indices were examined to determine whether the model aligned with the theoretical structure. It is stated that the fit indices should meet the acceptable or good fit values specified in the literature (Hu and Bentler, 1999; Marsh et al., 2004; Marsh et al., 2010; Kline, 2015). Accordingly, an acceptable fit value for *X*^2^/df is <5, and a good fit value is <2; for RMSEA, an acceptable fit value is <0.10, and a good fit value is <0.08; for CFI, IFI, GFI, AGFI, and TLI, an acceptable fit value is >0.90, and a good fit value is >0.95.

The results of the initial CFA analysis indicated that the *X*^2^/df and RMSEA values for the tested model exceeded the acceptable thresholds (*X*^2^/df: 3.62, RMSEA: 0.114, CFI: 0.909, GFI: 0.873, TLI: 0.874). If the fit indices from the first-order CFA analysis do not indicate an adequate fit, modification suggestions should be reviewed (MacCallum et al., 1992; Byrne, 2010; Seçer, 2015). Some semantic differences arising from translation or cultural adaptation may influence the relationships between items. In such cases, making theoretically appropriate and explainable modifications is crucial for the validity of the adapted scale (Cheung and Rensvold, 2002). For instance, Schumacker and Lomax (2010) advocate for the use of modifications when correlations between items are theoretically plausible. Due to the fit indices not falling within the reference value ranges, modification indices were examined, and covariance adjustments were made between the error terms of “V7-V11” and “V10-V2.” The low fit indices of the initial model indicated that theoretically meaningful relationships between certain items needed to be considered. These covariance modifications were not only data-driven but also theoretically justified. The relationship between item 7 (“I will try my best in this task”) and item 11 (“If I succeed in this task, I will feel proud of myself”) can be interpreted through Bandura’s (1997) theory of self-efficacy, which links effort with expected emotional outcomes such as pride or satisfaction. Similarly, Brown (2015) notes that correlating residuals is acceptable in CFA when items reflect related psychological constructs, especially when cultural interpretation may blur factor distinctions. In this case, participants may perceive exertion and pride as inherently connected components of situational motivation. After the first modification, the fit indices of the model were reexamined, revealing that the *X*^2^/df value was 3.18, RMSEA was 0.104, CFI was 0.925, GFI was 0.889, and TLI was 0.895. In the second modification, the relationship established between V1 (“I think I can overcome this task”) and V10 (“I believe anyone can perform this task well”) arises from the tendency to generalize the perception of confidence in task success. This correlation, reflecting participants’ general sense of confidence regarding the task, better represents the Probability of Success construct. While correlating error terms is sometimes viewed with caution, it is considered acceptable when supported by theoretical and semantic overlap (MacCallum et al., 1992; Cheung and Rensvold, 2002). For example, Bandura (1997) suggests that perceived self-efficacy and expected emotional outcomes—such as pride or satisfaction—often operate in tandem, which may cause overlapping item responses even in distinct sub-factors.

**Figure 2:**
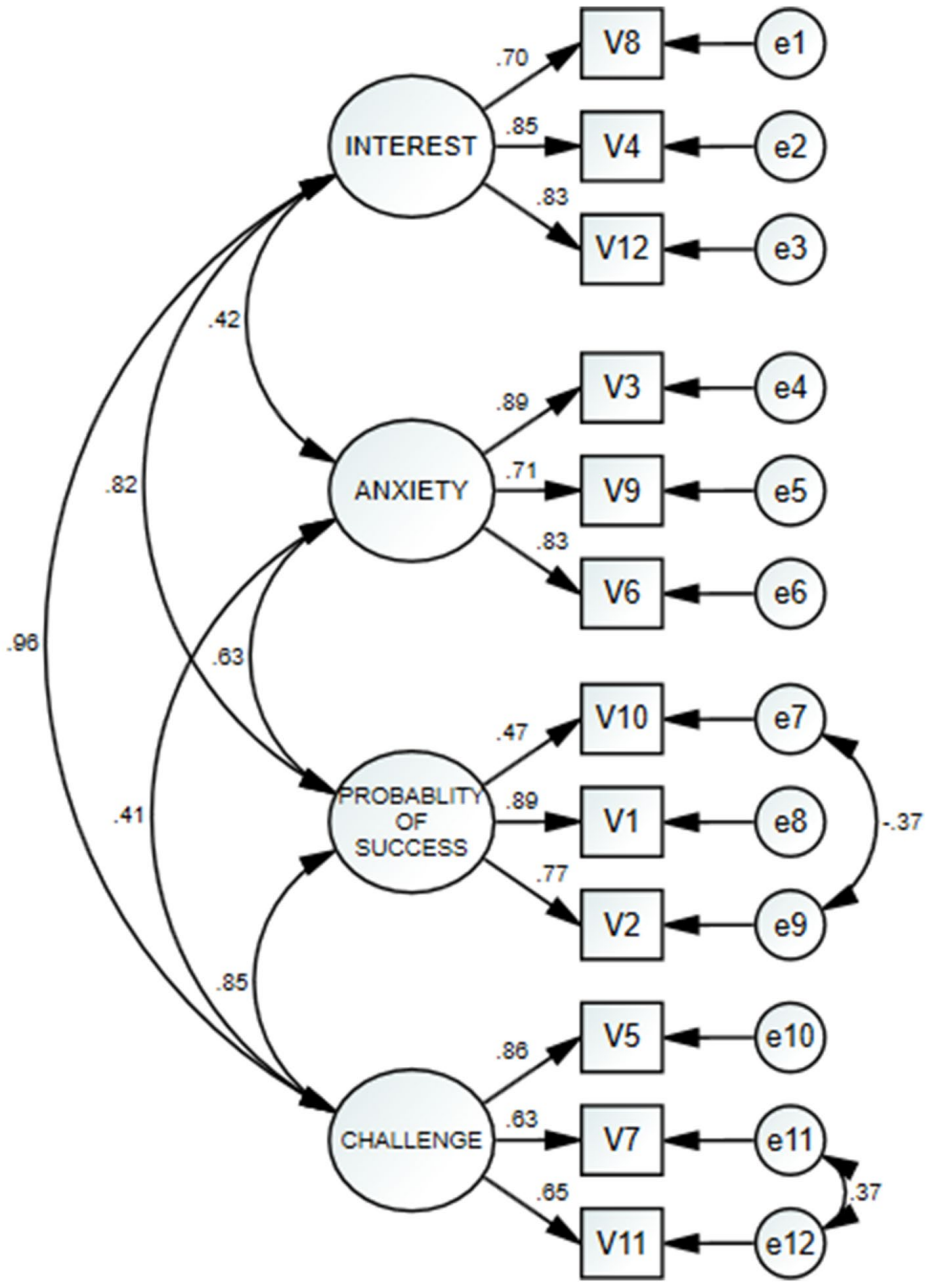
Results of CFA analysis for QCM.

Brown (2015) further emphasizes that in Confirmatory Factor Analysis, correlating error terms is justified when items are conceptually related or when cultural and linguistic adaptations result in minor shifts in item interpretation. Given that the Turkish context may frame certain motivational constructs differently, allowing for these modifications strengthens the model’s cultural fit without compromising its theoretical integrity. After the modification process, the model was rerun. Figure 2 presents the path diagram obtained after the modification. Upon examining the diagram, it was observed that each item’s factor loading met the requirement of being 0.30 or higher (Seçer, 2015). When the fit indices in Table 4 were reviewed, the *X*^2^/df value was 2.79, RMSEA was 0.094, CFI was 0.940, GFI was 0.905, and TLI was 0.914. These values indicate acceptable fit levels.

**Table 4:** Model-data fit values for the QCM.

*X*^2^/df	RMSEA	CFI	GFI	TLI
2.79	0.094	0.940	0.905	0.914

### 3.5 Reliability analysis

Internal consistency was assessed using Cronbach’s alpha (*α*), with a Cronbach’s alpha value of 0.70 or higher considered adequate and 0.80 or higher considered good internal consistency (Cronbach, 1951; George and Mallery, 2003). According to Table 5, the Cronbach’s alpha value was found to be 0.835 for the Interest factor, 0.837 for the Anxiety factor, 0.703 for the Success Probability factor, 0.802 for the Challenge factor, and 0.893 for the entire QCM.

**Table 5:** Internal reliability test results.

QCM and its factors reliability coefficient	Items	Cronbach’s alpha Turkish short form	Cronbach’s alpha long form	Cronbach’s alpha short form
Interest	3	0.835	0.82	0.78
Anxiety	3	0.837	0.79	0.81
Probability of success	3	0.703	0.72	0.85
Challenge	3	0.802	0.66	0.71
QCM total		0.893		

To examine the item discrimination of the QCM, the mean scores of the top and bottom 27% groups for each item were compared. An independent samples *t*-test was conducted to determine whether there was a statistically significant difference between the scores of the top and bottom 27% groups (Table 6).

**Table 6:** Results of item analysis.

Factors	Items	Corrected item-total correlation	Cronbach’s alpha if Item deleted	Top 27% *X_*	Bottom 27% *X_*	*t*
Interest	V8	0.667	0.806	6.20	2.72	22.99**
	V4	0.707	0.771	5.76	2.96	19.67**
	V12	0.730	0.738	5.76	2.37	23.31**
Anxiety	V3	0.753	0.736	6.44	3.20	25.53**
	V9	0.664	0.842	7.00	2.63	42.27**
	V6	0.723	0.758	7.00	3.48	27.31**
Probability of success	V10	0.348	0.795	6.65	4.09	28.60**
	V1	0.754	0.265	6.44	3.31	30.08**
	V2	0.505	0.633	6.74	3.81	24.00**
Challenge	V5	0.611	0.770	6.39	3.61	24.21**
	V7	0.662	0.717	6.87	3.83	19.80**
	V11	0.680	0.695	7.00	3.97	25.80**

**p < 0.001.

According to Table 6, the corrected item-total correlations of the QCM range from 0.348 to 0.754. Additionally, based on the results of the independent samples *t*-test conducted to examine the differences between the top and bottom 27% groups for each item, all items of the QCM were found to be statistically significant in terms of discrimination (*p* < 0.001). Furthermore, the mean differences for each item are positive. These results indicate that all items of the QCM exhibit high discriminatory power and that the QCM possesses strong psychometric properties.

## 4 Discussion and conclusions

This study aimed to adapt the QCM, originally shortened by Freund et al. (2011), into Turkish to assess motivation towards task-based learning. This study contributes a culturally adapted tool to measure situational motivation in Turkish educational settings. The Turkish adaptation of the QCM used in this study is a 7-point Likert-type scale consisting of 12 items. QCM includes four factors: “Interest,” “Anxiety,” “Challenge,” and “Success Probability.” In the Turkish version of the questionnaire, the distribution of items under the factors has undergone changes. Upon examining the item-total correlations, eight positive and four negative items were identified in the Turkish adaptation. Accordingly, the minimum score obtainable from the QCM is 36, while the maximum score is 84. This adaptation aligns with recommendations in the literature that emphasize culturally adapted instruments should retain both semantic and conceptual integrity (Tanzer, 2005).

The study began with translation procedures, followed by a correlation analysis to determine the relationship between the original and the Turkish versions of the QCM. According to the findings, the correlation coefficient between the original and Turkish versions was 0.931, which is very close to 1. The *p*-value for this correlation was 0.000, indicating statistical significance at the 0.01 level.

In the pilot phase of the study, data were analyzed to examine internal consistency and the compatibility of item-total correlations. The reliability analysis confirmed internal consistency, as indicated by Cronbach’s alpha values above acceptable thresholds. Following these analyses, the main applications were conducted, and a Confirmatory Factor Analysis (CFA) was performed for the Turkish adaptation of the QCM. As noted by George and Mallery (2003), values above 0.70 are generally accepted as indicators of good internal reliability, supporting the stability of measurement in psychometric studies.

In the context of descriptive statistics, Kaiser-Meyer-Olkin (KMO) and Bartlett’s tests were conducted to determine whether the sample size of the dataset was suitable for factor analysis. The results of the KMO and Bartlett’s tests indicated *p* < 0.001. The KMO value suggested adequacy of the sample size, as a KMO value of 0.70 or higher is desired (Seçer, 2015). The findings, with a KMO value exceeding 0.70, indicated that the dataset was appropriate for factor analysis (Field, 2009).

To test the construct validity of the QCM, a Confirmatory Factor Analysis (CFA) was conducted, and the obtained values (*X*^2^/df = 2.79, GFI = 0.905, CFI = 0.940, TLI = 0.914, and RMSEA = 0.094) supported the compatibility of the model with the theoretical structure. These results demonstrated that the Turkish version of the QCM measures consistently while preserving the original structure of the QCM. The findings were observed to fall within acceptable ranges (Pituch and Stevens, 2015; Hair et al., 1998; Byrne, 2010). Thus, the construct validity of the QCM was confirmed. This is consistent with the model fit criteria discussed by Hu and Bentler (1999), who argue that RMSEA values below 0.10 and CFI/TLI values above 0.90 indicate acceptable model fit. Moreover, beyond linguistic adaptation, ensuring conceptual and cultural equivalence is essential for cross-cultural validity. Tanzer (2005) emphasize that cross-cultural adaptation must address semantic, idiomatic, and experiential equivalence—not just language. For example, constructs such as “challenge” or “success probability” may carry different connotations in the Turkish educational context, where collective success expectations and uncertainty avoidance may influence interpretation (Van de Vijver and Leung, 1997). These cultural dimensions could shape how participants understand and respond to certain items, affecting the scale’s construct representation.

Additionally, the high factor loadings obtained in this study (ranging from 0.56 to 0.92) reflect a strong association between the items and their underlying latent constructs. As emphasized by Hair et al. (1998), this level of factor loading contributes significantly to construct validity. The overall reliability (Cronbach’s alpha = 0.893) further supports the internal consistency of the Turkish adaptation. Kline (2015) notes that such consistency values above 0.80 indicate a well-performing psychometric instrument, suitable for both research and applied settings.

The sub-dimension scores were as follows: 0.835 for Interest, 0.837 for Anxiety, 0.703 for Success Probability, and 0.802 for Challenge, indicating that the sub-dimensions measured consistently. The Cronbach’s alpha value calculated for the overall QCM was 0.893, strongly supporting the reliability of the QCM. These coefficients are consistent with established reliability benchmarks and prior adaptation studies (e.g., Karaman et al., 2023; Tavşancıl, 2002). These values obtained for the Turkish short form are generally consistent with the Cronbach’s alpha coefficients of the original short form. For instance, the Interest sub-dimension in the original short form had a coefficient of 0.78, while the Turkish short form calculated a slightly higher value of 0.835. Similarly, the Anxiety sub-dimension had a Cronbach’s alpha coefficient of 0.81 in the original short form, which increased to 0.837 in the Turkish version. However, for the Success Probability sub-dimension, the Turkish short form value (0.703) was slightly lower than that of the original short form (0.85). This difference may stem from the linguistic and cultural adaptation processes of the Turkish version. For the Challenge sub-dimension, the original short form coefficient was 0.71, whereas the Turkish short form recorded a higher value of 0.802.

The independent sample t-test results for the mean scores of the top and bottom 27% groups confirmed the item discrimination of the QCM. Statistically significant differences were found between the top and bottom groups for all items (*p* < 0.001). These results indicate that each item of the QCM effectively differentiates motivation levels and possesses sufficient discriminative power. These findings are consistent with Seçer (2015), who emphasized the importance of item discrimination analysis in scale validation studies in Turkish educational contexts. The Cronbach’s alpha values and high item discrimination characteristics of the Turkish adaptation of the questionnaire demonstrate its strong psychometric properties in terms of validity and reliability. Specifically, the acceptable reliability value observed for the Success Probability sub-dimension may be attributed to the effects of linguistic differences on perceptions of motivation. This aspect warrants further investigation through future studies with different samples.

These values are largely consistent with those reported in the original short form. Minor differences—such as the slightly lower reliability score in the Success Probability dimension—may be attributed to linguistic or contextual interpretations shaped by the Turkish educational environment.

Taken together, the validity and reliability results suggest that the adapted scale maintains the psychometric rigor of the original version and functions effectively in the Turkish educational context. In conclusion, the Turkish version of the QCM is a valid and reliable instrument for measuring pre-service teachers’ situational motivation. The findings demonstrate that the QCM makes a significant contribution to the Turkish literature on motivation measurement. These results align with prior findings by Freund et al. (2017) and Smith et al. (2020), who emphasized the importance of situational motivation measurement in diverse educational contexts. Similar CFA structures and acceptable fit thresholds were also observed in cross-cultural adaptations of motivation scales (Elford, 2022; Smith et al., 2020), supporting the structural robustness of the Turkish QCM adaptation. In particular, Elford (2022) demonstrated how contextual factors, such as AR environments, affect motivation, while our findings confirm the QCM’s ability to capture such context-driven motivation in outdoor task-based activities.

While the QCM was validated in an outdoor, task-based learning context using mobile devices, its applicability across different educational settings remains to be explored. Freund et al. (2017) highlighted that task-specific motivation can vary based on contextual factors such as environment and modality. Future research should examine whether the QCM demonstrates similar psychometric properties in traditional classroom environments or virtual settings, where motivational cues may differ significantly.

## 5 Limitations and future research

A convenient sampling method was employed in this study. The number of female students in the study group exceeded that of male students. This gender imbalance, along with the exclusive focus on pre-service biology and science teachers, may limit the generalizability of findings to other disciplines or more gender-balanced populations. Prior research suggests that motivation-related constructs may vary by academic domain and gender (Eccles and Wigfield, 2002). Future studies should validate the Turkish QCM across diverse teaching fields—such as humanities, mathematics, or language education—and in samples with more balanced gender representation. The QCM was administered in the context of task-based outdoor activities involving mobile devices for learning plant species. Therefore, it was applied specifically to science and biology pre-service teachers who had completed general biology coursework and laboratory training. This study is the first to examine the psychometric properties of the Turkish adaptation of the QCM. Future researchers using the Turkish adaptation of the QCM are encouraged to replicate similar studies, which could enhance the generalizability of the findings. Additionally, one limitation of the study is the lack of an equivalent QCM adapted into Turkish to measure pre-service teachers’ situational achievement motivation for similar QCM validity purposes. Another methodological limitation involves the correlating of error terms in the CFA model, which, while statistically improving model fit, requires a strong theoretical rationale. In the present study, modifications were guided not only by empirical fit indices but also by conceptual justification drawn from previous adaptation research (Cheung and Rensvold, 2002; Schumacker and Lomax, 2010). Moreover, cultural and linguistic nuances—such as interpretations of “pride” and “challenge”—may subtly influence item functioning. Therefore, future adaptation studies should integrate qualitative methods (e.g., cognitive interviews) to better capture such culturally grounded meaning variations.

## Data Availability

The datasets generated and analyzed during the current study are not publicly available due to ethical considerations and participant confidentiality, but are available from the corresponding author upon reasonable request.
